# Application of multi-scale feature extraction and explainable machine learning in chest x-ray position evaluation within an integrated learning framework

**DOI:** 10.1007/s00330-025-12097-9

**Published:** 2025-11-05

**Authors:** Chaowei Ma, Rui Peng, Bingjie Li, Dong Zhang, Rong Zhang, Zhiqing Zhang, Shaoyi Du, Yani Bai

**Affiliations:** 1 Department of Radiology, Xijing Hospital, Fourth Military Medical University, Xi’an, China; 2 State Key Laboratory of Human-Machine Hybrid Augmented Intelligence, and Institute of Artificial Intelligence and Robotics, Xi’an Jiaotong University, Xi’an, China; 3 Department of Ultrasound, the Second Affiliated Hospital of Xi’an Jiaotong University, Xi’an Jiaotong University, Xi’an, China

**Keywords:** Chest X-ray, Position, Machine learning, Quality control, Random forest

## Abstract

**Objectives:**

This study presents a novel deep learning-machine learning fusion network for quantitative and interpretable assessment of chest X-ray positioning, aiming to analyze critical factors in patient positioning layout.

**Materials and methods:**

In this retrospective study, we analyzed 3300 chest radiographs from a Chinese medical institution, collected between March 2021–December 2022. The dataset was partitioned into the XJ_chest_21 subset for training automated segmentation model and the XJ_chest_22 subset to validate three classification models: Random Forest Fusion Network (RFFN), Threshold Classification (TC), and Multivariate Logistic Regression (MLR). After automatically measuring five positioning indicators in the images, the data were input into the models to assess positioning quality. We compared the performance metrics of the three classification models, including AUC, accuracy, sensitivity, and specificity. SHAP (Shapley Additive Explanations) was utilized to interpret feature importance in the decision-making process of the RFFN model. We evaluated measurement consistency between the Automated Measurement Model (AMM) and radiologists.

**Results:**

U-net++ demonstrated significantly superior performance compared to U-net in multi-target segmentation accuracy (mean Dice: 0.926 vs. 0.812). The five positioning metrics showed excellent agreement between AMM and reference standards (r = 0.93). ROC analysis indicated that RFFN performed significantly better in overall image quality classification (AUC, 0.982; 95% CI: 0.963, 0.993) compared to both TC (AUC, 0.959; 95% CI: 0.923, 0.995) and MLR (AUC, 0.953; 95% CI: 0.933, 0.974).

**Conclusion:**

Our study introduces a novel segmentation-based random forest fusion network that achieves accurate image positioning classification and identifies critical operational factors. Furthermore, the clinical interpretability of the fusion model was enhanced through the application of the SHAP method.

**Key Points:**

***Question*** *How can AI-driven interpretable methods be utilized to assess patient positioning in chest radiography and enhance radiographers’ accuracy?*

***Findings*** *The Random Forest Fusion Network (RFFN) outperformed Threshold Classification (TC) and Multivariate Logistic Regression (MLR) in positioning classification (AUC = 0.98).*

***Clinical relevance*** *An integrated framework that combines deep learning and machine learning achieves accurate image positioning classification, identifies critical operational factors, enables expert-level image quality assessment, and delivers automated feedback to radiographers.*

**Graphical Abstract:**

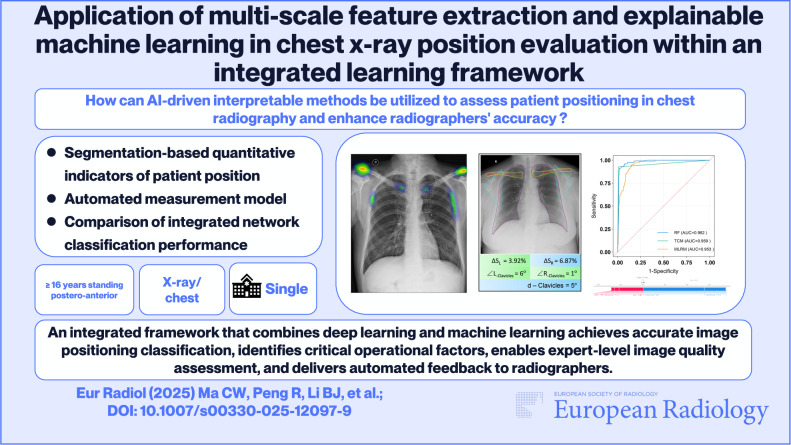

## Introduction

Chest X-ray is a common diagnostic examination, and the setting of the patient’s examination position is an important part of the radiographer’s work. Thus, real-time display of positioning features and accurate assessment during examinations are crucial for the radiographer to promptly correct positioning and potentially improve image quality. However, the accurate assessment of the position under manual operation has been facing many challenges. First, during the examination process, several factors may influence the technician’s assessment of the projection position, including the patient’s body type, level of cooperation, projection angle, disease, etc. A study involving 64,315 cases has shown that the majority of cases failed to meet at least one quality criterion [1]. Incorrect positioning of the patient can lead to superimposition of skeletal structures and distortion of anatomical projections in the image, which increases the burden of examination and the risk of misdiagnosis for the patient. Suboptimal positioning caused 46% of image rejections—the leading cause [2, 3]. Patient positioning errors led to 50–77% repeat exams [2–5]. Second, postural assessment is mostly a subjective evaluation based on visual analysis, which is based on officially published image quality standards [6–9], and is plagued by poor observer subjective consistency and non-quantitative evaluation criteria, which affects the mutual recognition of image postural evaluation [10]. Finally, the evaluation process requires high levels of concentration and is laborious and time-consuming.

In recent years, deep learning (DL) neural networks have been extensively utilized in medical image analysis, tackling a wide range of core tasks from disease diagnosis to image quality control (QC). Meng et al [11] incorporated quantitative indicators for positioning assessment as a key component of QC and found good correlation between such indicators and radiologists’ subjective evaluations. Through evaluation of critical anatomical alignment parameters (e.g., scapula/lung/clavicle alignment), quantitative methods offer real-time feedback to radiographers regarding positional adequacy, thereby reducing the gap between subjective visual inspection and objective quality metrics [12–14].

Machine learning has demonstrated significant predictive capabilities in decision-making tasks and has garnered increasing attention from clinicians. However, its “Black-box” nature poses challenges to interpretability—a crucial requirement in healthcare, where transparency and accountability are essential. Current studies [11, 13, 14] predominantly employ linear regression and threshold-based models for image positioning classification, offering operational simplicity within clinical workflows but exhibiting limitations in classification accuracy and consistency of threshold determination. To mitigate the “black-box” issue, the SHapley Additive exPlanation (SHAP) method provides a unified framework for interpreting machine learning model outputs, enabling visualization of individual feature contributions and enhancing understanding of model decisions [15].

This study proposes an integrated network classification approach that combines deep learning and machine learning techniques (Random Forest Fusion Network, RFFN), based on SHAP interpretability analysis. The primary objective is to develop and validate an interpretable classification model for assessing patient chest X-ray positioning, enabling real-time feedback on positioning decisions and assisting the radiographer in optimizing examination procedures. Furthermore, the study evaluates the classification performance of the multiple logistic regression (MLR) model, the threshold classification (TC) model and the RFFN model.

## Materials and methods

### Patients and study design

**Fig. 1:**
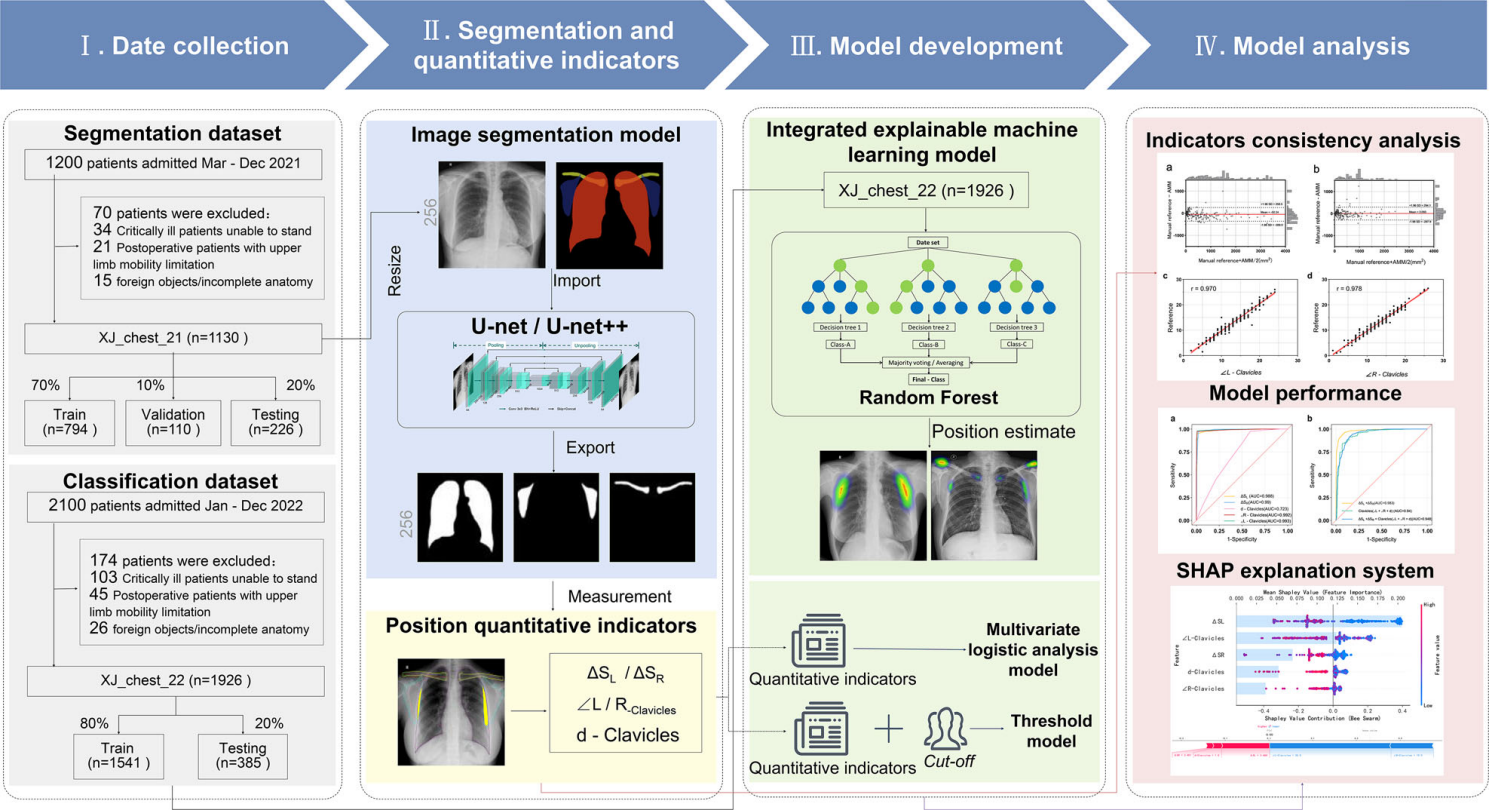
Study flowchart

This retrospective study was approved by the Ethics Review Committee of the Xijing Hospital of the Fourth Military Medical University (Approval numbers: KY20252147-C-1). This study aims to assess radiographer positioning in chest X-rays, provide real-time feedback on positioning decisions. The detailed workflow is provided in Supplementary Fig. S3. Chest X-ray data were randomly collected from Xijing Hospital between March 2021 and December 2022 for analysis. The inclusion criteria were patients aged ≥ 16 years who underwent standing postero-anterior chest radiography and could independently cooperate with standard postural maneuvers. Patients with emergencies or critical illnesses, those who had undergone thoracic surgical treatment, or those with limited upper-extremity mobility were excluded. This study focused on the quantitative evaluation and analysis of the characteristics of patient position layout in chest X-ray examinations and constructed an AI-based patient position evaluation model. Details of the study design are displayed in Fig. 1.

### Dataset and QC of image labels

#### Dataset construction

Using stratified random sampling, 3300 patients were recruited to construct two independent datasets of chest X-rays with varying quality levels. The dataset XJ_chest_21 consisted of 1130 cases after excluding 70 individuals (34 critically ill patients unable to stand, 21 postoperative patients with upper limb limitations, and 15 foreign objects/incomplete anatomy) from the 1200 samples collected between March and December 2021. The dataset XJ_chest_22 included 1926 cases after excluding 174 individuals (103 critically ill patients unable to stand, 45 postoperative patients with upper limb limitations, and 26 foreign objects/incomplete anatomy) from the 2100 samples collected between January and December 2022. Specifically, the XJ_chest_21 dataset (*n* = 1130) trained image segmentation and measurement algorithms, while XJ_chest_22 (*n* = 1926) developed an integrated AI model for classification positioning analysis.

#### Dataset image annotation and QC

Two radiologists (M.C.W., lead author, 15 years image QC; B.Y.N., Senior Chest Radiologist, 25 years) delineated XJ_chest_21 via *FACT 2.0* with polygon annotations for six radiographic projection boundaries—two for lung fields, two for scapulae, and two for clavicles—using standardized anatomical landmarks. They also conducted double-blind independent measurements on the test subset (*n* = 226) for five key anatomical parameters: the proportion of left/right scapula-lung overlap (ΔSL/ΔSR), clavicle tilt angles (∠L/R-Clavicles), and bilateral angle difference (d-Clavicles).

Three radiologists (M.C.W. and B.Y.N. from XJ_chest_21 delineation; Z.R., 13 years QC specialist) independently conducted binary positioning assessments (Pass/Fail) for XJ_chest_22 QC. These binary assessments included not only the overall evaluation of image positioning quality but also the ground truth labels for 5 quantitative indicators: ΔSL, ΔSR, ∠L-Clavicles, ∠R-Clavicles, and d-Clavicles. Discrepancies among the three radiologists were resolved through majority voting, whereby the assessment supported by at least two radiologists was established as the reference standard.

Two-level hierarchical reviews ensured quality control for both datasets: First-level screening excluded 70 (XJ_chest_21) and 174 (XJ_chest_22) cases based on patient eligibility and image quality. Second-level refinement differed by dataset: XJ_chest_21 underwent anatomical boundary audits (6 structures: 2 lung fields, 2 scapulae, 2 clavicles) and corrected images with wrongly labeled boundaries; XJ_chest_22 used three radiologists (B.Y.N./M.C.W./Z.R.) for binary positioning assessments (Pass/Fail) and resolved the discrepancy in the true image results through voting (*n* = 208) to ensure label consistency. Quantitative annotations by radiologists were independent of hierarchical reviews: anatomical parameter measurements on XJ_chest_21’s test subset (*n* = 226) began only after its two-level review.

### Quantitative indicators and measurements

A review of guidelines and research results related to chest X-ray image quality [6, 9, 16–18] reveals that, in the overall assessment of image quality, the main challenges affecting patient position are as follows: (1) the levelness and symmetry of bilateral clavicles; (2) the non-overlapping of scapulae and lung fields. Therefore, in this study, the aspects with low satisfaction in the above two position layouts were selected as the focus of patient position evaluation, and five quantitative indicators were calculated: ΔS_L_/ΔS_R_, ∠L/R-Clavicles and d-Clavicles.

Manual quantitative measurements were performed on XJ_chest_21’s test subset (*n* = 226) to validate model measurement accuracy. Both manual and automated measurements employed identical methodologies: two radiologists independently conducted triplicate measurements for each image, with results averaged to establish the reference standard.

The calculation method for ΔS_L/R_ is presented in Formula (1). If the shoulder blades do not overlap with the lung field, the ΔS_L/R_ value is defined as 0 (Supplementary Fig. S1a). The ∠L/R-Clavicles is defined in Supplementary Fig. S1b. The d-clavicle is calculated as the absolute value of the difference in inclination angle between the left and right clavicles, Formula (2).

1 $$\Delta {{\rm{S}}}_{{\mbox{L}}}/_{{\mbox{R}}}=\frac{{{\rm{S}}}_{{{\mbox{L}}}/{\mbox{R}}-{\mbox{Lung}}}\cap {{\rm{S}}}_{{{\mbox{L}}}/{\mbox{R}}-{\mbox{Scapulae}}}}{{{\rm{S}}}_{{{\mbox{L}}}/{\mbox{R}}-{\mbox{Lung}}}}$$

2 $${{\rm{d}}}-{{\rm{Clavicles}}}=\,{{{\rm{| }}}\angle {{\rm{L}}}}_{-{{\rm{Clavicles}}}}-{\angle {{\rm{R}}}}_{-{{\rm{Clavicles}}}}{{\rm{| }}}$$

### Deep learning model training

#### Image preprocessing

The XJ_chest_21 dataset was randomly partitioned into a training set (*n* = 791), a validation set (*n* = 113), and a test set (*n* = 226) at an approximate ratio of 7:1:2. Prior to training, the images underwent pixel resizing (256*256) and format normalization. Regarding the XJ_chest_22 dataset, 80% was employed for model training, while 20% served as test data. Five quantitative postural indicators were automatically extracted via the segmentation model pre-trained on XJ_chest_21, serving as independent variables (*x*). The actual QC outcomes (pass = 1, fail = 0), determined by radiologists’ binary assessments, were utilized as dependent variables (*y*).

#### Segmentation models

We trained two multi-output segmentation models based on U-net and U-net++ without pre-trained weights. The U-net-based model comprises three parallel encoders and a shared decoder. Feature fusion is achieved via skip connections to preserve detailed information. The U-net++based segmentation model extracts multi-scale features from the input image using an encoder equipped with components like convolutional blocks and batch normalization layers. The decoder’s up-sampling operation and feature processing mirror those of the U-net-based model. However, U-net++ employs a nested skip-connection mechanism to fuse feature maps across different levels, preventing information loss and outputting three independent segmentation maps for the scapula, lungs, and clavicle.

During the training phase, we employed a combination of Binary Cross-Entropy (BCE) loss and Dice loss(Dice) as the loss function (3):

3 $$L\left(t,p\right)=0.5\,\times {BCE}\left(t,p\right)+0.5\,\times {Dice}(t,p)$$

Here, *t* and *p* represent the target and predicted results, respectively. We optimized the network using an Adam optimizer with a learning rate of 0.001. The training process was configured with a batch size of 4 and 50 epochs. Both segmentation models underwent 5-fold cross-validation on the training set.

#### Position classification model

For position evaluation and classification, the image segmentation-based fusion network model employs a two-stage joint architecture. In the first stage, the automated measurement model segments the anatomical regions of the image and computes quantitative indicators. Subsequently, the output of the measurement model serves as the input for the classification model to predict and classify the image’s posture. The classification task of the fusion network utilizes a machine-learning RF model. After cross-validation optimization, the optimal parameter combination (n_ estimators = 300, max_ depth = 5) is selected.

To compare the performance of the RFFN model, two image segmentation-based quantitative network models are established as baseline models: the TC model and the MLR model. The TC model calculates the classification thresholds for each postural quantitative metric based on the actual results. An image is deemed “pass” if all its indicators are below the thresholds; otherwise, it is “fail.” The thresholds are determined as follows: an independent ROC analysis is conducted on the quantitative indicators from the XJ_chest_22 dataset, the Youden’s index is calculated, and the corresponding cutoff value is chosen as the optimal classification threshold. The MLR model takes each quantitative metric as an independent variable (*x*) and the actual postural outcome as a dependent variable (*y*). The corresponding probabilities of the classification results are calculated using the fitted formula.

### Evaluation of model performance

#### Automated measurement model (AMM)

In this study, the DL-based AMM is tasked with two key operations: image segmentation and metric quantification. Consequently, we assess the algorithm’s performance from these two perspectives by employing a diverse set of metrics. To evaluate the model’s segmentation capabilities, we incorporate metrics such as Pixel Accuracy (PA), Precision, Recall, F1-score, Mean Dice (mDice), and Mean Intersection over Union (mIoU). The corresponding calculation formulas are presented in Supplementary Table S1. The accuracy of the quantitative indicators serves as a further means to validate the segmentation model. We utilize the Bland–Altman test to evaluate the consistency in the measurement of quantitative indicators between the model and radiologists.

#### Classification model

We conducted training and validation of the fusion network and quantization network on the XJ_chest_22 dataset. Subsequently, the optimized model was evaluated on the test set. To gauge the model’s effectiveness, we computed and analyzed several performance metrics, namely accuracy, specificity, recall, F1-score, AUC-PR, and the area under the ROC curve (AUC).

#### Explainability of model

This study utilizes the SHAP method to evaluate feature importance and interpret the classification outcomes of the RFFN model. The approach provides insights from two complementary perspectives: (1) Global interpretation assesses the overall contribution of each key feature to positioning evaluation, including their interaction effects; (2) Local interpretation visualizes the distribution of SHAP values for individual samples, thereby elucidating the model’s decision-making process in specific cases.

### Statistical analysis

We employed the scikit-learn library and SHAP in Python 3.2 and GraphPad Prism 10.1 (GraphPad Software Inc.) for statistical analysis. The performance of models for each classification task was evaluated using ROC curve analysis and the corresponding AUC. The intraclass correlation coefficient (ICC) was used to assess the inter-rater reliability among radiologists. An ICC value greater than 0.85 was indicative of good consistency. Bland–Altman plots and linear correlation plots were used to illustrate the reliability of the model against the reference standard and the discrepancies in measurements. Statistical significance was defined as a two-sided *p*-value less than 0.05.

## Results

### Patient characterization and position assessment

In this study, we constructed two chest X-ray image datasets. Manual annotation was carried out in accordance with the patient position QC principles, and the evaluation results are presented in Table 1. The findings indicated that, in both datasets, the overall non-compliance rate of image positions attributable to lung field overlap and clavicle unevenness exceeded 75%.

**Table 1:** Basic characteristics of the dataset images

Variable	XJ_Chest_21				XJ_Chest_22		
	Training	Validation	Testing	Total	Training	Testing	Total
Number of images, *n*	794	110	226	1130	1541	385	1926
Male, *n* (%)	397 (50)	57 (51.8)	107 (47.35)	561 (49.6)	596 (38.7)	136 (35.3)	732 (38)
Age, years^a^	50 (37–60)	52.5 (35–61)	51.5 (37–61)	50 (37–60)	50 (36–59)	50 (36–60)	50 (36–59)
Case type							
Normal, *n* (%)	150 (18.9)	25 (22.7)	53 (23.5)	228 (20.2)	320 (20.8)	70 (18.2)	390 (20.2)
Pneumonia, *n* (%)	230 (29.0)	30 (27.3)	50 (22.1)	310 (27.4)	455 (29.5)	109 (28.3)	564 (29.3)
Rib fracture, *n* (%)	135 (17.0)	22 (20.0)	40 (17.7)	197 (17.4)	259 (16.8)	66 (17.1)	325 (16.9)
Pleural effusion, *n* (%)	85 (10.7)	10 (9.1)	29 (12.8)	124 (11.0)	210 (13.6)	51 (13.2)	261 (13.6)
Other pathologies, *n* (%)	194 (24.4)	23 (20.9)	54 (23.9)	271 (24.0)	297 (19.3)	89 (23.2)	386 (20.0)
Physical characteristics							
Obesity (BMI ≥ 30), *n* (%)	120 (15.1)	20 (18.2)	35 (15.5)	175 (15.5)	280 (18.2)	66 (17.1)	346 (18.0)
Normal (18.5 ≤ BMI < 30), *n* (%)	510 (64.2)	68 (61.8)	145 (64.2)	723 (64.0)	913 (59.2)	239 (62.1)	1152 (59.8)
Thin (BMI ≤ 18.5), *n* (%)	164 (20.7)	22 (20.0)	46 (20.4)	232 (20.5)	348 (22.6)	80 (20.8)	428 (22.2)
Screening scenarios							
Outpatient screening, *n* (%)	400 (50.4)	57 (51.8)	101 (44.7)	558 (49.4)	817 (53.0)	207 (53.8)	1024 (53.2)
Physical examination, *n* (%)	235 (29.6)	29 (26.4)	89 (39.3)	353 (31.2)	443 (28.8)	104 (27.0)	547 (28.4)
Inpatient follow-up, *n* (%)	159 (20.0)	24 (21.8)	36 (16.0)	219 (19.4)	281 (18.2)	74 (19.2)	355 (18.4)
Body position characteristics							
LLF-NOP, *n* (%)	438 (55.2)	53 (48.2)	90 (39.9)	581 (51.4)	779 (50.6)	156 (40.5)	935 (48.5)
RLF-NOP, *n* (%)	550 (69.3)	76 (69.1)	145 (64.2)	771 (68.2)	1034 (67.1)	242 (62.8)	1276 (66.3)
Lung field pass, *n* (%)	397 (50.0)	46 (41.8)	81 (35.9)	524 (46.4)	697 (45.2)	142 (36.9)	839 (43.6)
LC level, *n* (%)	528 (66.5)	74 (67.3)	167 (73.9)	769 (68.1)	1024 (66.5)	267 (69.4)	1291 (67.0)
RC level, *n* (%)	562 (70.8)	80 (72.7)	179 (79.2)	821 (72.7)	1154 (77.9)	305 (79.2)	1459 (75.8)
Clavicle bilateral symmetry, *n* (%)	652 (82.1)	87 (79.1)	184 (81.4)	923 (81.7)	1214 (66.5)	307 (79.7)	1521 (79.0)
Clavicle pass, *n* (%)	408 (51.4)	59 (53.6)	131 (58.0)	598 (53.0)	817 (53.0)	214 (55.6)	1031 (53.5)
Image overall pass, *n* (%)	209 (26.3)	27 (24.5)	43 (19.0)	279 (24.7)	385 (25.0)	69 (18.0)	454 (23.6)
Image overall fail, *n* (%)	585 (73.7)	83 (75.5)	183 (81.0)	851 (75.3)	1156 (75.0)	316 (82.0)	1472 (76.4)

Except for age (median), other categorical values are presented as number (percentage)

*LLF-NOP* left lung field no-overlap present, *RLF-NOP* right lung field no-overlap present, *LC* left clavicle, *RC* right clavicle

^a^ Data: medians, (interquartile ranges)

### Deep learning segmentation model screening

In this study, we compared two DL models, U-net and U-net++, for boundary segmentation. Segmentation performance is presented in Table 2, indicating that U-net++ outperforms U-net overall and offers more consistent results in multi-class segmentation. The segmentation results of the two models are shown in the Supplementary Fig. S2. Evidently, U-net++ accurately detects segmentation landmarks, particularly in the clavicle and scapula regions, with remarkable pixel-level classification ability. Thus, we chose U-net++ for boundary detection and subsequent analysis of the anatomical regions of the patient’s body position.

**Table 2:** The segmentation results of the U-net and U-net++

Model	Organs	PA	mIoU	mDice	Precision	Recall	F1 Score
U-net	Scapula	0.988	0.775	0.872	0.839	0.907	0.872
	Lung	0.950	0.617	0.756	0.718	0.789	0.752
	Clavicle	0.992	0.679	0.808	0.588	0.942	0.724
U-net++	Scapula	0.987	0.857	0.922	0.918	0.930	0.922
	Lung	0.951	0.952	0.975	0.983	0.970	0.975
	Clavicle	0.993	0.786	0.879	0.857	0.906	0.879

*PA* pixel accuracy, *mIoU* mean intersection over union, *mDice* mean dice coefficient

### Reliability analysis of quantitative indicators

Table 3 presents the reliability analysis results of quantitative image indicators from the XJ_Chest_21 test set, as evaluated by two radiologists and a measurement model. This analysis encompasses inter-observer consistency and the measurement deviation between the model and the reference standard.

**Table 3:** Analysis of the measurement results and consistency of quantitative indicators between observers and the AMM

Indicators	Inter-observer				Manual reference vs. AMM			
	Radiologist 1	Radiologist 2	ICC (95% CI)	*p*-value*	Reference	AMM	r (95% CI)	*p*-value
∠L-Clavicles, (°)	13.33 ± 4.72	13.72 ± 4.73	0.965 (0.949–0.975)	< 0.001	13.57 ± 4.68	13.54 ± 4.27	0.97 (0.961–0.977)	< 0.001
∠R-Clavicles, (°)	12.69 ± 4.59	12.45 ± 4.64	0.972 (0.962–0.979)	< 0.001	12.57 ± 4.58	12.77 ± 4.35	0.978 (0.972–0.983)	< 0.001
ΔS_L_	0.031 ± 0.041	0.028 ± 0.042	0.963 (0.946–0.974)	< 0.001	0.029 ± 0.04	0.027 ± 0.038	0.864 (0.825–0.894)	< 0.001
ΔS_R_	0.017 ± 0.023	0.015 ± 0.023	0.943 (0.923–0.957)	< 0.001	0.016 ± 0.023	0.016 ± 0.022	0.893 (0.862–0.917)	< 0.001

*ICC* intraclass correlation coefficient, *AMM* automated measurement model, *ΔSL* left scapula-lung overlap ratio, *ΔSR* right scapula-lung overlap ratio, ∠*L-Clavicle* left clavicle tilt angle, ∠*R-Clavicle* right clavicle tilt angle

* Significance obtained using the *F*-test for truth value 0. (°): degrees; Radiologist 1: M.C.W.; Radiologist 2: B.Y.N.

**Fig. 2:**
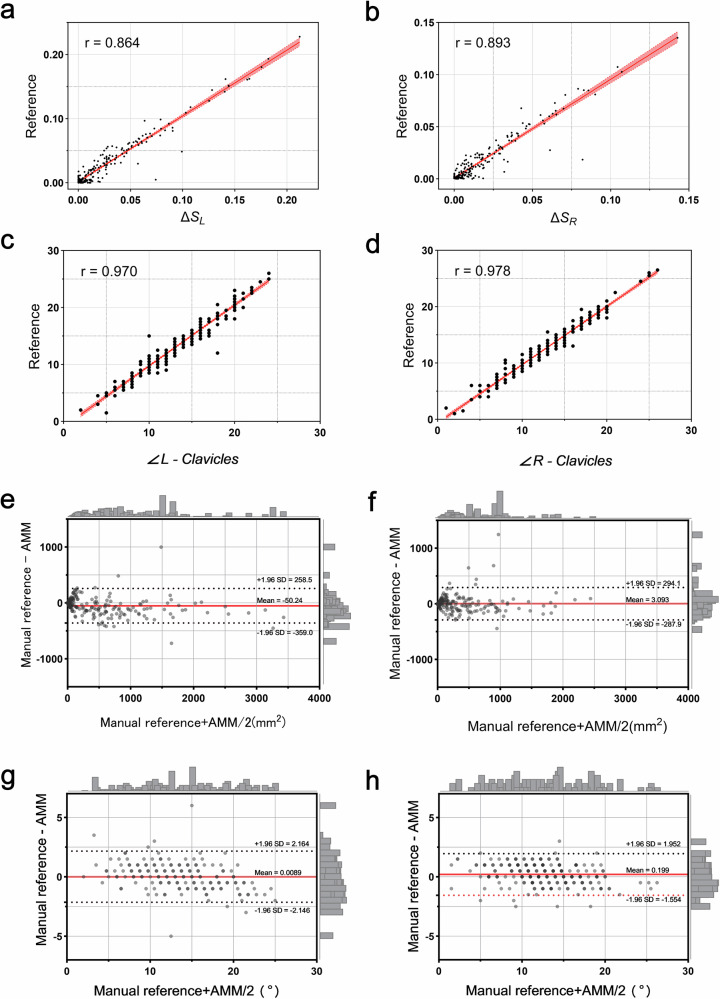
Scatter plot (**a**–**d**) showing the correlation between AI model quantitative indicators (**a** ΔS_L_, **b** ΔS_R_, **c** ∠L-Clavicles, **d** ∠R-Clavicles) measurements and reference standard values, with the *x*-axis denoting the AI model and the *y*-axis representing the manual reference standard. Bland–Altman plots (**e**–**h**) depict the discrepancies between AMM (automated measurement method) and manual reference standards for four indices: (**e** ΔS_L_, **f** ΔS_R_, **g** ∠L-Clavicles, **h** ∠R-Clavicles); the *x*-axis represents the reference values, while the *y*-axis denotes AMM measurements, with 95% limits of agreement (1.96 SD) demarcating upper/lower bounds. Most data points for all four metrics lie within these limits, demonstrating good agreement between AMM and manual measurements

The measurements from the radiologists demonstrated excellent internal consistency, with a mean ICC of 0.961 (95% CI: 0.945–0.971). A linear correlation was found between the manual reference standard and the model’s measurements (Fig. 2a–d). High correlations were observed for the left and right clavicle angles (r = 0.970 and 0.978, respectively), followed by the overlap ratios of the scapula and lung fields (r = 0.864 and 0.893, respectively). The measurement model achieved high precision across all four indicators.

The Bland–Altman plot illustrates the measurement deviation between the manual reference standard and AMM. The mean differences in the overlap areas of the scapula and lung fields were −50.24 mm² on the left (−359 to 258.2 mm²) and 3.09 mm² on the right (−287.9 to 294.1 mm²). For the clavicle angles, the mean differences were 0.009° on the left (−2.146 to 2.164°) and 0.199° on the right (−1.554 to 1.952°). These results indicate a high level of agreement between the model-based and manual measurement methods (Fig. 2e–h).

Inter-rater agreement among three physicians demonstrated near-perfect reliability (Fleiss’ Kappa = 0.856, 95% CI 0.831–0.881) for binary classification of 1926 chest radiographs (XJ_Chest_22), with 89.2% observed concordance.

### Analysis of the position classification model

#### Threshold setting of the TC model and MLR model

**Fig. 3:**
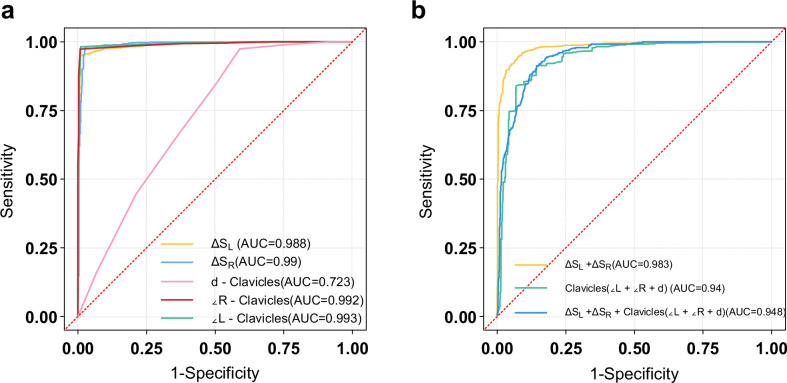
**a** ROC curves for univariate quantitative indicators (ΔSL, ΔSR, ∠L-Clavicles, ∠R-Clavicles, d-Clavicles) of the TC model, **b** ROC curves for combined variables in the MLR

Figure 3 and Table 4 present the results of univariate ROC analysis and MLR model analysis of the image quantification indicators for the training set (*n* = 1541) of the XJ_Chest_22 dataset. The optimal threshold values corresponding to the maximum Youden’s index of individual quantification indicators are detailed in Table 4. Based on the critical values of the five position quantification indicators, we constructed the final TC model. In the MLR model based on quantification indicators, the Hosmer-Lemeshow test indicated that the overall image evaluation model had the best goodness of fit (*p* = 0.7356). Specifically, the formulas for the proportion of scapula and lung field overlap (${\hat{y}}_{1}$), clavicle level (${\hat{y}}_{2}$), and overall image QC (${\hat{y}}_{3}$) are as follows:

$${\hat{y}}_{1}=5.64\,-\,0.925{\Delta S}_{L}-0.731{\Delta S}_{R}$$

$${\hat{y}}_{2}=10.02-0.424\,{\angle L}_{-{Clavicles}}\,-0.176{\angle R}_{-{Clavicles}}\,-0.892\,{d}_{-{Clavicles}}\,$$

$${\hat{y}}_{3}= \,	7.22-\,0.52\,{\Delta S}_{L}-0.44{1\Delta S}_{R}-0.29{\angle L}_{-{Clavicles}}\,-0.094{\angle R}_{-{Clavicles}}\, \\ 	-0.483{d}_{-{Clavicles}}$$

**Table 4:** The results of the TC model univariate ROC analysis and MLR model analysis of the image quantification indicators

Methods	Quantification indicators	AUC (95% CI)	Accuracy	Sensitivity	Specificity	Cutoff
Univariate	ΔS_L_	0.988 (0.982–0.993)	0.957	0.951	0.990	4.3
	ΔS_R_	0.990 (0.984–0.996)	0.972	0.977	0.974	4.45
	∠L-Clavicles	0.993 (0.989–0.996)	0.984	0.981	0.990	14.5
	∠R-Clavicles	0.992 (0.988–0.995)	0.978	0.973	0.992	14.5
	d-Clavicles	0.723 (0.698–0.749)	0.683	0.974	0.41	4.5
Combined variable	ΔS_L_ + ΔS_R_	0.983 (0.977–0.988)	0.934	0.945	0.924	N/A
	Clavicles(∠L + ∠R + d)	0.940 (0.928–0.953)	0.882	0.840	0.931	N/A
	ΔS_L_ + ΔS_R_ + Clavicles(∠L + ∠R + d)	0.948 (0.937–0.958)	0.885	0.912	0.856	N/A

*ΔSL* left scapula-lung overlap ratio, *ΔSR* right scapula-lung overlap ratio, ∠*L-Clavicle* left clavicle tilt angle, ∠*R-Clavicle* right clavicle tilt angle, *d-Clavicles* bilateral clavicle angle difference, *Cutoff* cutoff value

#### Comprehensive evaluation of classification models

**Fig. 4:**
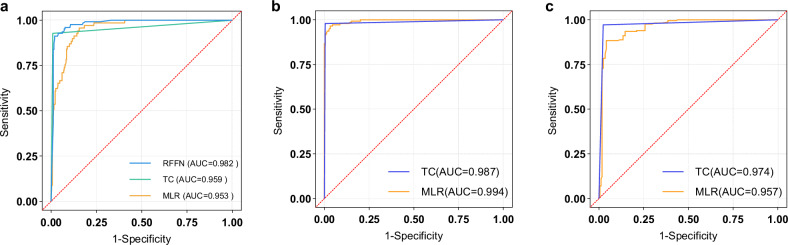
**a** The ROC curves for the overall image classification efficiency of three models (RFFN, TC, MLR). **b**, **c** Comparison of the ROC curves between the TC and MLR models in evaluating **b** scapula-lung field overlap and **c** clavicle angle assessment

The classification performance of three models was validated on XJ_Chest_22 test set, with multiple performance indicators calculated (Table 5). The RFFN model demonstrated the optimal classification efficiency for overall image classification (*p* < 0.001), followed by the TC model, which outperformed the MLR model (*p* = 0.009) (Fig. 4a). In scapula-lung field overlap classification(using variables ΔSL and ΔSR), the MLR model (AUC = 0.994) outperformed the TC model (AUC = 0.987); in clavicle angle assessment(using variables ∠L-Clavicles, ∠R-Clavicles, and d-Clavicles), the TC model (AUC = 0.974) showed superiority over the MLR model (AUC = 0.957) (Fig. 4b, c).

**Table 5:** The overall classification performance indicators of the three models in terms of patient position

Model	AUC	Accuracy	Recall	Specificity	AUC-PR	F1 score-max
TC	0.959 (0.923–0.995)	0.979 (0.959–0.993)	0.928 (0.839–0.976)	0.991 (0.973–0.998)	0.915 (0.822–0.961)	0.941
MLR	0.953 (0.933–0.974)	0.899 (0.820–0.927)	0.865 (0.839–0.891)	0.783 (0.748–0.883)	0.800 (0.689–0.878)	0.756
RFFN	0.982 (0.963–0.993)	0.938 (0.909–0.959)	0.873 (0.824–0.923)	0.904 (0.865–0.944)	0.946 (0.890–0.975)	0.838

*AUC* area under the curve, *AUC-PR* area under the curve of precision-recall

#### Explainability of the RFFN model

**Fig. 5:**
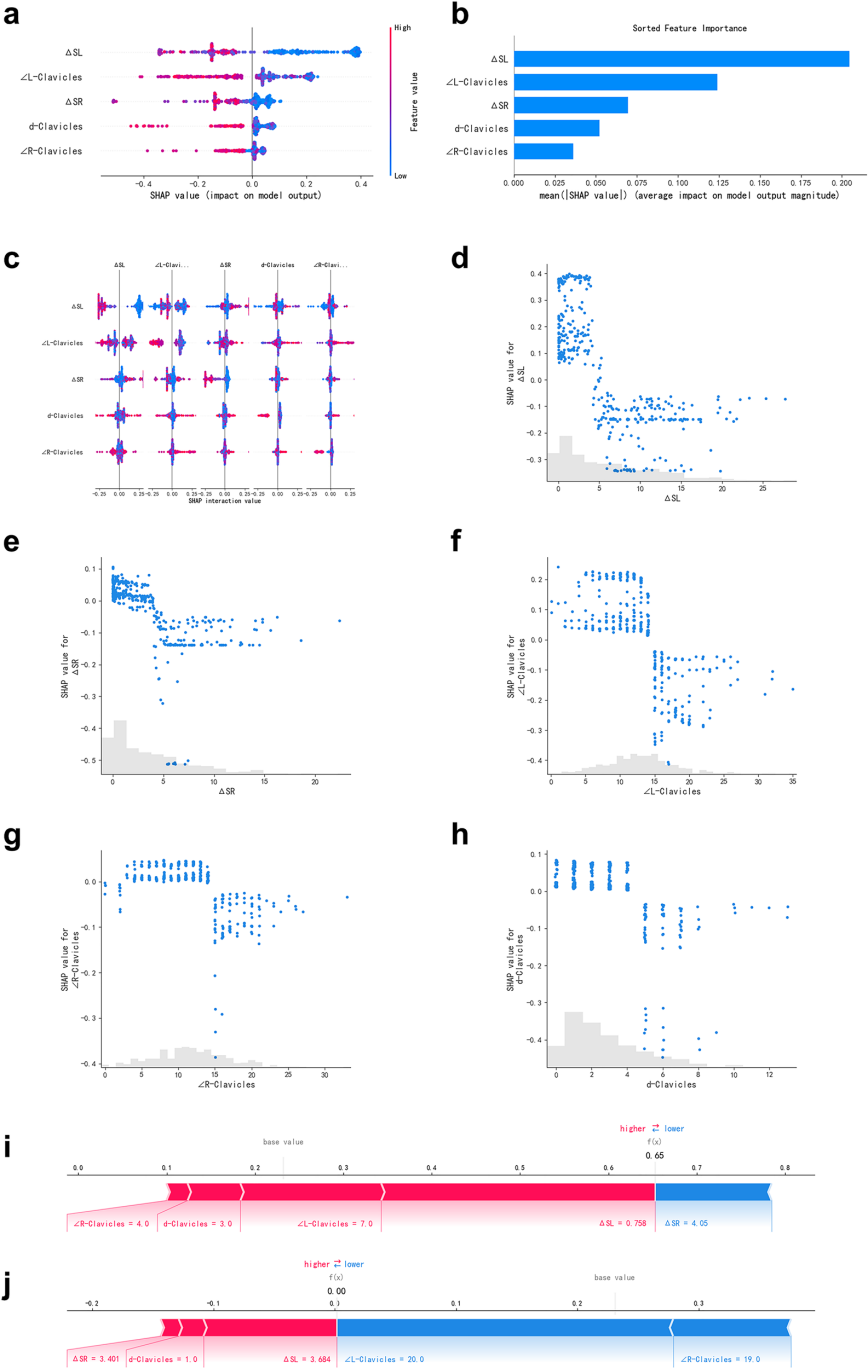
This figure presents an interpretability analysis of the RFFN results using the SHAP method. **a**, **b** The overall contribution of each feature to the model’s decision-making process. The dependence plots (**c**) reveal the interdependencies among the features within the model. **d**–**h** SHAP scatter plots, which uncover the nonlinear relationships between five individual features and their corresponding SHAP values. Positive SHAP values indicate alignment with the “pass” classification category in the model. Force plots (**i**, **j**) depict the directional contributions of two representative sample features. The blue features on the right are those that push the posture toward the “fail” category, while the red features on the left are those that push the posture toward the “pass” category. Sample **i** (Predicted probability: 0.65; Classified as “pass”): The first four features, characterized by high values, collectively enhance the prediction confidence, whereas a low ΔSR value exerts an inhibitory effect. Sample **j**: The elevated ΔSR and ΔSL values (3.401 and 3.684, respectively) contribute positively to the prediction. However, ∠L-Clavicles and ∠R-Clavicles have negative contributions. Despite favorable bilateral clavicle symmetry, the final model output f(x) > 0 resulted in a classification of “failure”

Feature importance quantification (Fig. 5a, b) ranked global contributions using mean absolute SHAP values (descending order). ΔSL emerged as the most critical feature (mean: 0.21), demonstrating dominant influence on postural assessment. ∠L-Clavicles and ΔSR constituted the core feature set (means: 0.122 and 0.063, respectively), collectively driving model decisions. Contributions from d-Clavicles and ∠R-Clavicles were marginal (means: 0.051 and 0.036). The self-interaction effect (Fig. 5c) of ΔSL shows a broad distribution (± 0.25), indicating a strong nonlinear influence on the model output. In contrast, ∠L-Clavicles exhibits a marginal interaction effect close to zero (± 0.1). A pronounced synergistic interaction between ΔSL and ∠L-Clavicles was identified as the most significant feature of interdependence.

#### Error analysis of the TC model

Notably, 4.94% (19/385) of cases exhibited clavicle angle discrepancies exceeding the predefined threshold (4.5°) but were manually rated as acceptable by radiologists. As shown in Table S2, this discrepancy primarily occurred in the narrow angular range of 0° < ∠L/∠R < 6°, where the mean d-clavicle was 4.8 ± 0.2°. This indicates that the model’s sensitivity (97.4%) may overestimate clinical significance in narrow angular ranges.

## Discussion

This study evaluated the accuracy of a deep learning-machine learning fusion network for patient positioning assessment in chest X-ray. Three key findings were observed: First, U-Net++ achieved superior anatomical segmentation accuracy compared to U-Net. Second, AMM based on precise segmentation showed strong consistency with the radiologists’ positioning indicators. Third, the fusion network incorporating random forest outperformed the TC and MLR models in positioning classification, demonstrating better performance (AUC = 0.98) and objective interpretability.

Chest radiography generates projections of overlapping attenuation information after X-ray penetration of the human body [19]. This characteristic significantly increases the complexity and difficulty of disease diagnosis. Optimal patient positioning is critical for image interpretation [20] because positional deviations may lead to repeated exposures, disease misdiagnosis, and increased radiation doses. Consequently, the ability to obtain diagnostic-quality images through a single exposure has become a key indicator for evaluating radiographers’ professional competence. Prior studies on chest X-ray QC have revealed substantial discrepancies between routine clinical chest radiographs and international guidelines [8, 9, 15]. Specifically, only 1.1–4% of images met the guidelines’ criteria for overall image quality, while patient positioning indicators showed 13% of images with scapulae outside the lung fields and 42% with symmetric clavicle alignment [17, 18]. In contrast, this study demonstrated scapular and clavicle compliance rates of 43.6% and 53.5%, respectively, suggesting potential advantages in the examination protocols or operator training employed. Nevertheless, further optimization remains necessary. Enhancing the single-examination success rate remains a long-standing challenge, while establishing an interpretable evaluation framework to assist operators serves as a critical solution. Objective and reproducible quantitative indicators, corresponding to radiologists’ subjective visual perception, serve as the foundation of QC procedures [8, 15], thereby establishing a connection between image representation and result interpretability.

In the domain of chest X-ray QC, DL models have demonstrated significant performance across various tasks. Specifically, DenseNet121 attained an AUC of 0.93 in classification [14]. ResNet-34 achieved anatomical positioning consistency ranging from 94 to 98% at a 4-mm threshold [11]. U-Net yielded Dice coefficients between 0.93 and 0.96 for lung segmentation [21], and the dual-channel CNN model SDFN reached a lung region identification accuracy of 95.84% [22]. However, current studies often encounter limitations related to dataset size and heterogeneity, with most focusing on single-task performance and lacking sufficient clinical validation of quantitative metrics. This study experimentally demonstrates that U-Net++, through its nested skip connections that enhance multi-scale feature fusion, significantly improves anatomical structure segmentation accuracy (mDice: 0.926 vs. U-Net: 0.812). Furthermore, the proposed AMM exhibited strong agreement with manual measurements performed by senior radiologists ($\bar{r}$ = 0.93), thereby contributing novel evidence for high-precision quantitative assessment in chest X-ray QC.

Most mainstream AI-assisted detection models employ end-to-end DL approaches, which are highly dependent on large-scale professional datasets [23–26]. This study constructed a patient positioning evaluation framework transitioning from quantitative analysis to qualitative assessment—providing a more robust and interpretable solution for image quality monitoring—whose proposed AI-integrated workflow for automated positioning assessment is detailed in Supplementary Fig. S3. Although existing guidelines have not established standardized thresholds for quantitative indicators, experts in different regions can formulate differentiated classification criteria based on clinical practice. The threshold models developed in this study for the scapula-lung field overlap ratio (AUC = 0.987) and clavicle angle (AUC = 0.974) also demonstrated favorable classification efficacy. It should be noted that the threshold models involve an uncertainty factor; the end evaluation results can be adjusted to meet specific requirements by modifying the threshold parameters. This characteristic increases the flexibility of model application to a certain extent but may also lead to subjective discrepancies in evaluation outcomes.

**Fig. 6:**
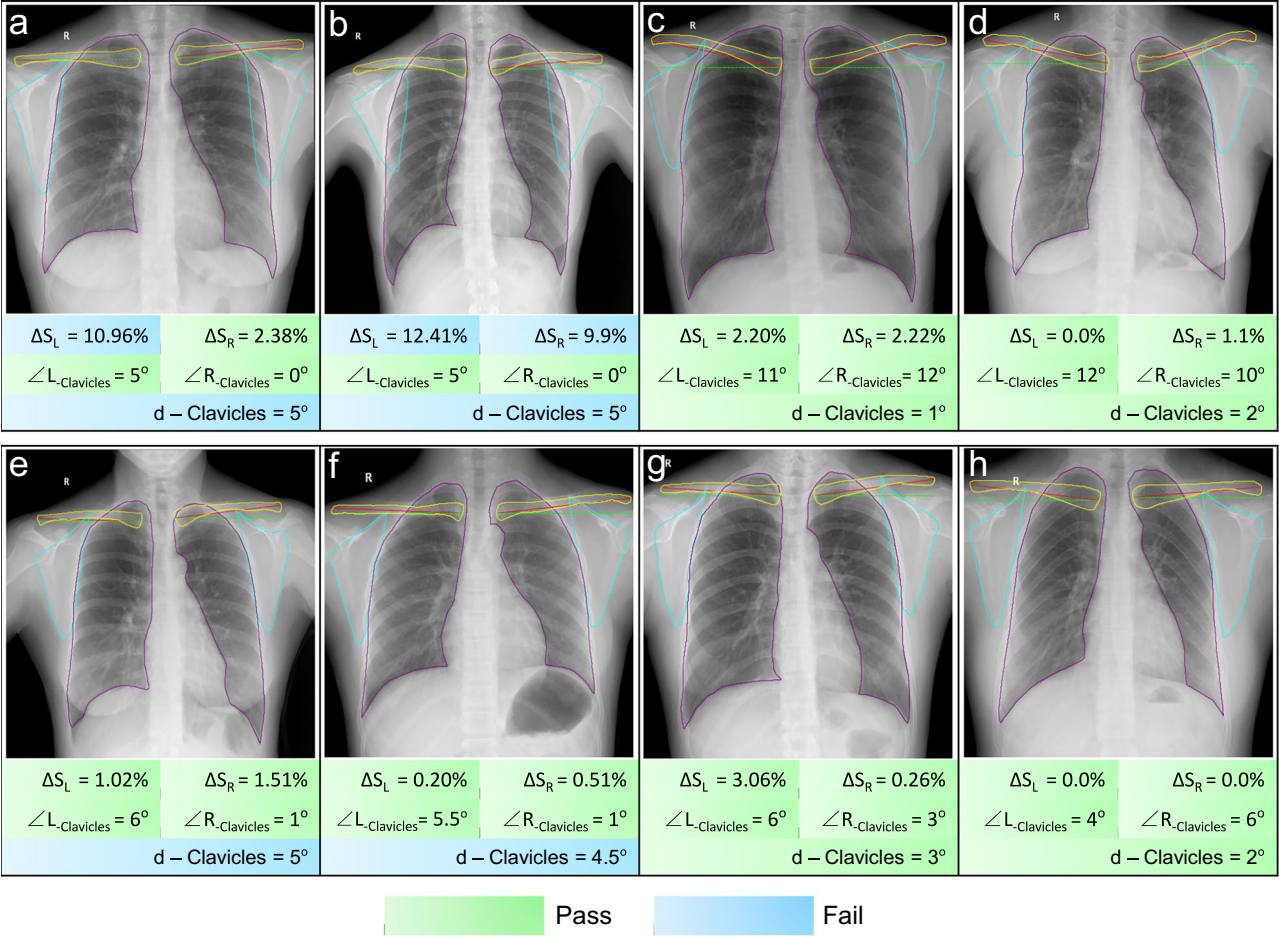
The evaluation results of the TC model’s independent quantitative indicators. **a**, **b** The ground truth for bilateral clavicle assessment is “pass,” but the TC model classifies them as “fail” due to d-Clavicles > 4.5°, compounded by scapula-lung field overlap. **c**, **d** Despite clavicle angles exceeding 6°, the model passed these cases as angles remained below the 14.5° threshold and d-Clavicles was minimal (< 4.5°), indicating good symmetry. **e**, **f** A d-Clavicles > 4.5° was the sole indicator for model failure, whereas clinicians passed these cases. **g**, **h** Clinically excellent positioning met all guideline criteria, with all metrics within normal ranges. When clavicle angles fall within 0–6°, model failures triggered by d-Clavicles > 4.5° diverge from clinical evaluations, highlighting d-Clavicles as a primary source of disagreement

In patient-positioning classification tasks, it is equally urgent to reduce the threshold bias caused by subjective evaluation fluctuations and sample limitations. A multi-stage hierarchical modeling approach achieved superior performance through task decomposition. This study employed a three-tier segmentation quantification classification framework to enable the analysis of positioning features. The results of the overall classification efficiency showed that the comprehensive performance of the RFFN model was superior (Table 5). Independent variable analysis of the threshold model revealed that d-Clavicles demonstrated high sensitivity (0.97), but low specificity (0.41), AUC (0.72), and accuracy (0.68). Notably, all models showed that this metric only exhibited high sensitivity for predicting outcomes. To investigate this further, we examined cases where clavicle angle discrepancies exceeded the critical threshold of 4.5°, yet were still classified as pass. Our analysis indicated that when both clavicle angles fell within a narrow angular range (0° < ∠L/R < 6°), discrepancies surpassing the predefined threshold (4.5°) were visually imperceptible to observers. Clinically, such cases are generally considered acceptable (Fig. 6). This observation is consistent with the ACR Chest Imaging Guidelines [8], which emphasize ‘visual symmetry assessment’ over rigid numerical thresholds for minor angular differences. The model’s strict adherence to the 4.5° cutoff may result in false-negative classifications within the 0–6° range. Therefore, in practical applications, threshold models should incorporate additional angular-range constraints to enhance interpretability and reduce misclassification.

In comprehensive comparisons of the three models, the RFFN demonstrated superior overall performance, followed by MLR. RFFN offer two key advantages: first, their variable importance analysis robustly identifies the classification weights of covariates while achieving optimal classification efficacy; second, under identical variable modeling conditions, RF models exhibit 50% lower classification error rates and greater robustness than logistic regression [27]. We integrated RF characteristics and radiomics to create a framework that pinpoints key classification indicators, guiding radiographers to optimize protocols and prevent poor outcomes.

The Interpretability framework based on SHAP effectively uncovers the “black box” nature of the RFFN, transforming its decision-making logic into clinically actionable insights. ΔSL (mean SHAP = 0.21) has the highest impact on postural assessment, confirming that “overlap of scapula and lung field” is the main indicator of non-standard postures. The secondary contributions of ∠L-Clavicles (0.122) andΔSR (0.063) reflect that the position of the left clavicle and scapula is a key factor affecting position. This result suggests that radiologists should give priority to the position of the scapula, especially the left side, when positioning. The feature dependence analysis within the model also indicates that the self-effect of ΔSL has a strong nonlinear influence on the model output. Among the characteristic interactions, the most significant one is the strong synergy between ΔSL and ∠L-Clavicles. Compared with TC, the SHAP scatter plot (Fig. 5d–h) reveals that RFFN recognizes nonlinear thresholds, while MLR oversimplifies the interaction to an additive effect (AUC decrease: 0.98 vs. 0.95). Meanwhile, the force plot of a single sample (Fig. 5i, j) indicates the features that are helpful for posture decision-making.

Limitations of this study include: (1) validation focused solely on patient positioning effects without accounting for equipment parameters and technical factors; (2) case selection was limited by sample size and diversity, with exclusion of images featuring severe cardiopulmonary diseases, feature loss, or foreign object interference to improve detection accuracy. (3) This study employed selected body position indicators to validate the fusion model. Future research will integrate all body position features and multi-center datasets to further assess the model’s reliability.

In this study, we developed a segmentation-based random forest fusion network evaluation system for image-positioning classification. This system demonstrates notable advantages in quantitative index analysis and QC classification performance, while mitigating concerns regarding the “black box” nature of machine learning through the application of the SHAP method. The AI-based approach not only achieves expert-level image quality management but also provides a novel approach to offer automated feedback to radiographers.

## Supplementary information

Supplementary information

## Abbreviations

AMM: Automated measurement model
BCE: Binary cross-entropy
ICC: Intraclass correlation coefficient
MIoU: Mean intersection over union
MLR: Multivariate logistic regression
PA: Pixel accuracy
QC: Quality control
RF: Random forest
SHAP: Shapley additive explanations
TC: Threshold classification
