# Supplementary material for: Application of multi-scale feature extraction and explainable machine learning in chest x-ray position evaluation within an integrated learning framework
Source: Eur Radiol. 2025 Nov 5;36(4):3143–57. doi: 10.1007/s00330-025-12097-9 (PMC13035635; doi:10.1007/s00330-025-12097-9)
Supplement: Supplementary file 1 — Supplementary information [file 330_2025_12097_MOESM1_ESM.pdf]

Application of multi-scale feature extraction and explainable machine learning in chest x-ray position evaluation within an integrated learning framework

Electronic Supplemental Material

Supplementary Table S1. Evaluation metrics

Evaluation Metrics	Note
TP	True Positive: A true positive sample which is classified as positive
FP	False Positive: A true negative sample which is classified as positive
TN	True Negative: A true negative sample which is classified as negative
FN	False Negative: A true positive sample which is classified as negative
Dice	$Dice = \frac{2 \times TP}{2 \times TP + FP + FN}$
mDice	$mDice = \frac{1}{n+1}\sum_{i=0}^{n} Dice$
IoU	$IoU = \frac{TP}{TP + FN + FP}$
mIoU	$mIoU = \frac{1}{n+1}\sum_{i=0}^{n} IoU$
PA	$PA = \frac{Total\ numbel\ of\ correctly\ classified\ pixels}{Total\ number\ of\ pixels}$
Precision	$Precision = \frac{TP}{TP + FP}$
Recall	$Recall = \frac{TP}{TP + FN}$
F1 Score	$F1\ Score = 2 \times \frac{Precision \times Recall}{Precision + Recall}$
FPR	$FPR = \frac{FP}{FP + TN}$
TPR	$TPR = \frac{TP}{TP + FN}$
ROC	The ROC curve : A plot of the TPR against the FPR at various threshold settings
AUC	AUC：Area Under the Curve

Supplementary Table S2. Distribution of clavicle angle discrepancies and the TC model-physician pass rate in the XJ_Chest_22 test set.

Angle Range	n	∠L (mean ± SD)	∠R (mean ± SD)	d-clavicle (mean ± SD)	Model Pass Rate (%)	Physician Pass Rate (%)
All cases	385	12.2°±5.0°	10.8°±4.9°	2.9°±2.4°	15.6	17.9
0° <∠L/∠R< 6°	33	3.8°±2.2°	2.7°±2.1°	2.9°±1.8°	36.4	84.5
Subgroup: d-clavicle >4.5°	19	3.8°±2.3°	2.6°±2.7°	4.8°±0.2°	0	89.5

Table Notes:

1. **Dataset specification:** All data were obtained from the XJ_Chest_22 test set.
2. **Definition of pass rate:** The "Pass Rate" refers to the percentage of cases categorized as "PASS" by either the TC model or three independent radiologists.
3. **Clinical correlation:** In the d-clavicle >4.5° subgroup, the model's 0% pass rate contrasts with the physicians' 89.5% pass rate, aligning with ACR guidelines [3], which prioritize visual symmetry assessment over rigid numerical thresholds for d-clavicle.

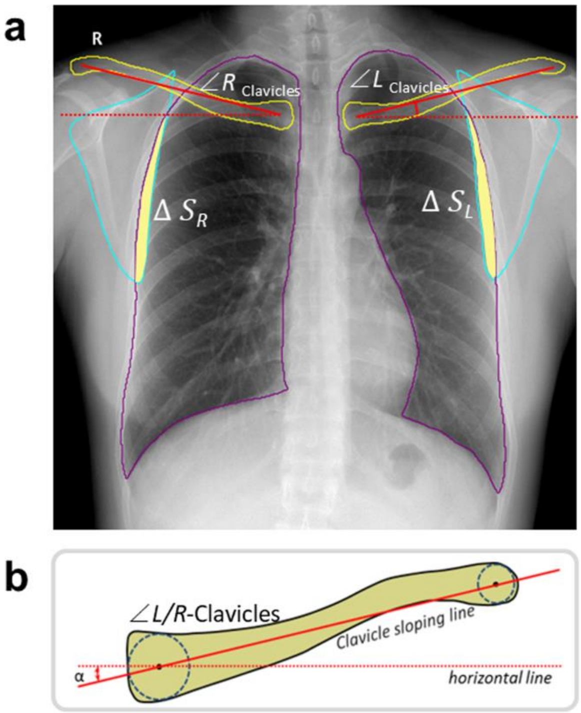

Figure S1 presents (**a**) a calculation diagram of quantitative metrics and (**b**) a schematic diagram of clavicle tilt angle.

The proportion of the overlapping area was determined by dividing the overlapping area of the scapula and lung field on the same side by the area of that the lung field. If the scapula does not overlap with the lung field, the value of $\Delta S_{L/R}$ is 0 (**Supplementary Fig.S1a**). The clavicle tilt angle (∠L/R-Clavicles) is defined as the angle between the horizontal line of the image and the line connecting the centers of the largest circles that are tangent to the inner boundaries at the two ends of the clavicle (**Supplementary Fig.S1b**). The d-Clavicles were calculated as the absolute value of the difference between the left and right clavicle tilt angles.

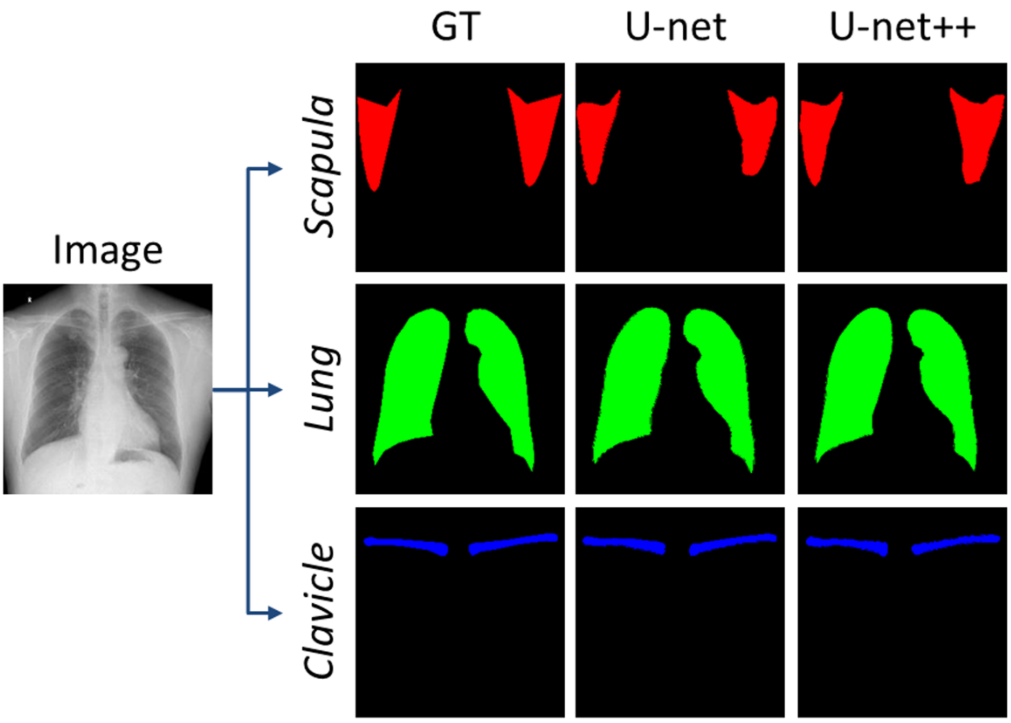

Figure S2 Segmentation result images of the U-net and U-net++.

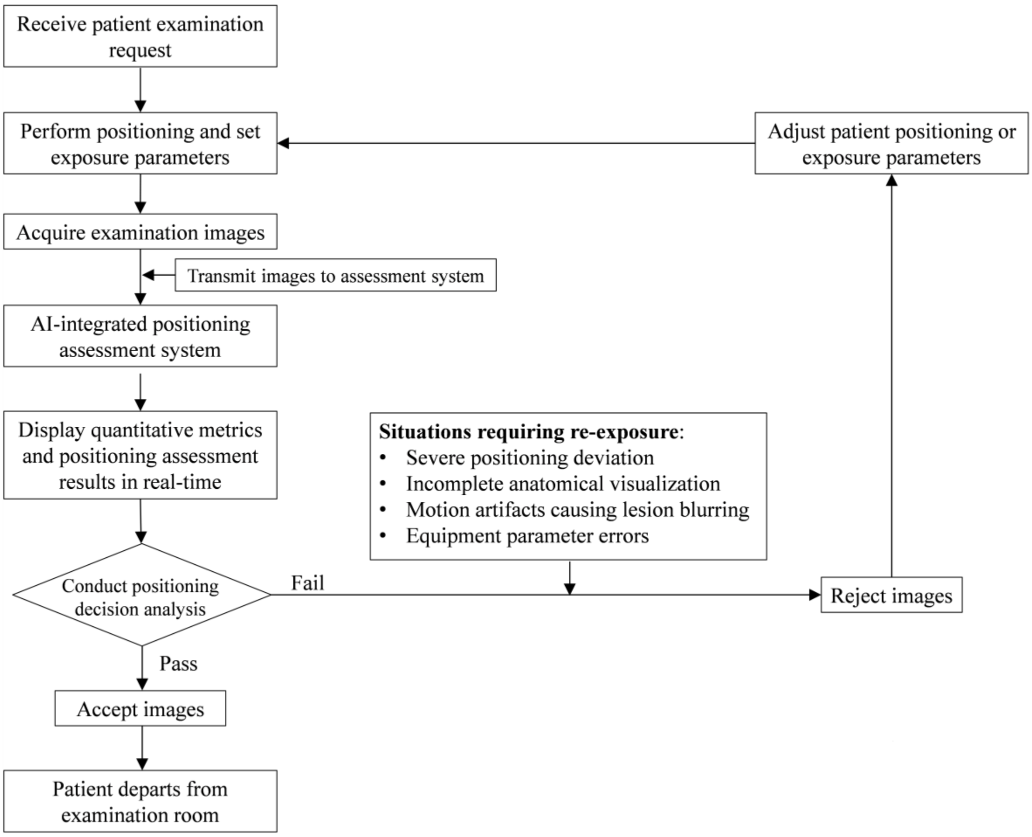

Figure S3: *AI-integrated Workflow Diagram for Automated Positioning Assessment in X-ray Examinations.*

SUPPLEMENTARY MATERIALS B – CODE

```
import numpy as np
from sklearn.ensemble import RandomForestRegressor
from sklearn.model_selection import GridSearchCV
from sklearn.model_selection import RandomizedSearchCV
import pandas as pd
import matplotlib.pyplot as plt
from sklearn.pipeline import Pipeline
plt.rcParams['font.family'] = 'sans-serif'
plt.rcParams['font.sans-serif'] = ['SimHei']
plt.rcParams['axes.unicode_minus'] = False

#回归问题评价指标
from sklearn.metrics import r2_score
from sklearn.metrics import mean_squared_error
from sklearn.model_selection import cross_val_score
#分类问题评价指标
from sklearn.metrics import accuracy_score
from sklearn.metrics import precision_score
from sklearn.metrics import recall_score
from sklearn.metrics import f1_score
from sklearn.metrics import confusion_matrix
from sklearn.metrics import roc_auc_score
from sklearn.metrics import classification_report
from sklearn.metrics import log_loss#对数损失（交叉熵损失）

X=pd.read_excel('samples.xlsx').values
y=pd.read_excel('result.xlsx').values.ravel()

# 将数据分为训练集和测试集
from sklearn.model_selection import train_test_split
X_train, X_test, y_train, y_test = train_test_split(X, y, test_size=0.2,
random_state=42)
print('X_train.shape',X_train.shape)
print('X_test.shape',X_test.shape)
print('y_train.shape',y_train.shape)
print('y_test.shape',y_test.shape)


from scipy.stats import uniform, randint
```

```
pipe=Pipeline([('rf', RandomForestRegressor())])
param_distributions = {
    'rf__n_estimators': randint(low=50, high=200),
    'rf__max_depth': randint(low=5, high=20),
    'rf__min_samples_split': randint(low=2, high=10),
    'rf__min_samples_leaf': randint(low=1, high=5),
    'rf__max_features': uniform(low=0.5, high=1) if 'max_features' in ['sqrt', 'log2'] else [None],
}
random_search = RandomizedSearchCV(pipe, param_distributions, n_iter=10, cv=5,verbose=1)
random_search.fit(X_train,y_train)

#获取最佳参数
best_params=random_search.best_params_
print("网格搜索的最佳参数：",best_params)

best_model = random_search.best_estimator_

from sklearn.metrics import mean_squared_error, mean_absolute_error

y_pred_train = best_model.predict(X_train)
y_pred_test = best_model.predict(X_test)

# 计算训练集的评价指标
r2_score_train = best_model.score(X_train, y_train)
mse_train = mean_squared_error(y_train, y_pred_train)
rmse_train = np.sqrt(mse_train)  # 计算 RMSE
mae_train = mean_absolute_error(y_train, y_pred_train)
mape_train = np.mean(np.abs((y_train - y_pred_train) / y_train)) * 100

# 计算测试集的评价指标
r2_score_test = best_model.score(X_test, y_test)
mse_test = mean_squared_error(y_test, y_pred_test)
rmse_test = np.sqrt(mse_test)  # 计算 RMSE
mae_test = mean_absolute_error(y_test, y_pred_test)
mape_test = np.mean(np.abs((y_test - y_pred_test) / y_test)) * 100

# 打印所有评价指标
print("训练集评价指标：")
print("R2:", r2_score_train)
print("MSE:", mse_train)
print("RMSE:", rmse_train)
print("MAE:", mae_train)
```

```
print("MAPE:", mape_train, "%")
print("\n 测试集评价指标：")
print("R2:", r2_score_test)
print("MSE:", mse_test)
print("RMSE:", rmse_test)
print("MAE:", mae_test)
print("MAPE:", mape_test, "%")


# 绘制训练集预测结果对比图
plt.figure()
plt.plot(range(1, X_train.shape[0] + 1), y_train, 'r-*', label='真实值')
plt.plot(range(1, X_train.shape[0] + 1), y_pred_train, 'b-o', label='预测值')
plt.xlabel('预测样本')
plt.ylabel('预测结果')
title_str = f"训练集预测结果对比\nRMSE={np.sqrt(mse_train)}"
plt.title(title_str)
plt.xlim([1, X_train.shape[0]])
plt.legend()
plt.grid()

# 绘制测试集预测结果对比图
plt.figure()
plt.plot(range(1, X_test.shape[0] + 1), y_test, 'r-*', label='真实值')
plt.plot(range(1, X_test.shape[0] + 1), y_pred_test, 'b-o', label='预测值')
plt.xlabel('预测样本')
plt.ylabel('预测结果')
title_str = f"测试集预测结果对比\nRMSE={np.sqrt(mse_test)}"
plt.title(title_str)
plt.xlim([1, X_test.shape[0]])
plt.legend()
plt.grid()

plt.show()


import seaborn as sns
from sklearn import metrics

# 创建一个包含训练集和测试集真实值与预测值的数据框
data_train = pd.DataFrame({
    'True': y_train,
    'Predicted': y_pred_train,
    'Data Set': 'Train'
```

```
})

data_test = pd.DataFrame({
    'True': y_test,
    'Predicted': y_pred_test,
    'Data Set': 'Test'
})

data = pd.concat([data_train, data_test])

# 自定义调色板
palette = {'Train': '#b4d4e1', 'Test': '#f4ba8a'}

# 创建 JointGrid 对象
plt.figure(figsize=(8, 6), dpi=1200)
g = sns.JointGrid(data=data, x="True", y="Predicted", hue="Data Set", height=10, palette=palette)

# 绘制中心的散点图
g.plot_joint(sns.scatterplot, alpha=0.5)
# 添加训练集的回归线
sns.regplot(data=data_train, x="True", y="Predicted", scatter=False, ax=g.ax_joint, color='#b4d4e1', label='Train Regression Line')
# 添加测试集的回归线
sns.regplot(data=data_test, x="True", y="Predicted", scatter=False, ax=g.ax_joint, color='#f4ba8a', label='Test Regression Line')
# 添加边缘的柱状图
g.plot_marginals(sns.histplot, kde=False, element='bars', multiple='stack', alpha=0.5)

# 添加拟合优度文本在右下角
ax = g.ax_joint
ax.text(0.95, 0.1, f'Train $R^2$ = {r2_score_train:.3f}', transform=ax.transAxes, fontsize=12,
                    verticalalignment='bottom', horizontalalignment='right', bbox=dict(boxstyle="round,pad=0.3", edgecolor="black", facecolor="white"))
ax.text(0.95, 0.05, f'Test $R^2$ = {r2_score_test:.3f}', transform=ax.transAxes, fontsize=12,
                    verticalalignment='bottom', horizontalalignment='right', bbox=dict(boxstyle="round,pad=0.3", edgecolor="black", facecolor="white"))
# 在左上角添加模型名称文本
ax.text(0.75, 0.99, 'Model = RF', transform=ax.transAxes, fontsize=12,
                    verticalalignment='top', horizontalalignment='left', bbox=dict(boxstyle="round,pad=0.3", edgecolor="black", facecolor="white"))
```

```
# 添加中心线
ax.plot([data['True'].min(),            data['True'].max()],            [data['True'].min(), data['True'].max()], c="black", alpha=0.5, linestyle='--', label='x=y')
ax.legend()
plt.savefig("TrueFalse.pdf", format='pdf', bbox_inches='tight')


from sklearn.model_selection import cross_val_score, KFold

def cv(best_model,X,y):
    # 设置交叉验证的折数
    kf = KFold(n_splits=5, shuffle=True, random_state=42)

    # 进行交叉验证并计算均方根误差（RMSE）
    rmse_scores = []
    r2_scores=[]
    for train_index, test_index in kf.split(X):
        X_train, X_test = X[train_index], X[test_index]
        y_train, y_test = y[train_index], y[test_index]

        best_model.fit(X_train, y_train)
        y_pred = best_model.predict(X_test)

        rmse = np.sqrt(mean_squared_error(y_test, y_pred))
        rmse_scores.append(rmse)

        r2=r2_score(y_test, y_pred)
        r2_scores.append(r2)

    # 输出 R2 分数的平均值和标准差
    print("R2 分数平均值: ", np.mean(r2_scores))
    print("R2 分数标准差: ", np.std(r2_scores))

    # 输出 RMSE 的平均值和标准差
    print("RMSE 平均值: ", np.mean(rmse_scores))
    print("RMSE 标准差: ", np.std(rmse_scores))

cv(best_model,X_train,y_train)


pip install shap==0.42.1

import shap
```

```
x_columns = ['n1', 'n2', 'n3','n4','n5']

explainer = shap.TreeExplainer(best_model['rf'])

# 计算 SHAP 值
shap_values = explainer.shap_values(X_test)

# 可视化 SHAP 值
shap.summary_plot(shap_values, X_test,feature_names=x_columns)


print("shap 维度:",shap_values.shape)
print("测试集维度:",X_test.shape)


shap.summary_plot(shap_values, X_test, plot_type="bar", show=False,feature_names=x_columns)
plt.title('Sorted Feature Importance')
plt.tight_layout()


# 创建主图
fig, ax1 = plt.subplots(figsize=(10, 8), dpi=1200)
shap.summary_plot(shap_values,X_test, x_columns, plot_type="dot", show=False, color_bar=True)
plt.gca().set_position([0.2, 0.2, 0.65, 0.65])  # 调整图表位置，留出右侧空间放热度条
# 获取共享的 y 轴
ax1 = plt.gca()
# 创建共享 y 轴的另一个图，绘制特征贡献图在顶部 x 轴
ax2 = ax1.twiny()
shap.summary_plot(shap_values,X_test, x_columns, plot_type="bar", show=False)
plt.gca().set_position([0.2, 0.2, 0.65, 0.65])  # 调整图表位置，与蜂巢图对齐
# 在顶部 X 轴添加一条横线
ax2.axhline(y=19, color='gray', linestyle='-', linewidth=1)  # 注意 y 值应该对应顶部
# 调整透明度
bars = ax2.patches  # 获取所有的柱状图对象
for bar in bars:
    bar.set_alpha(0.2)  # 设置透明度
# 设置两个 x 轴的标签
ax1.set_xlabel('Shapley Value Contribution (Bee Swarm)', fontsize=12)
```

```
ax2.set_xlabel('Mean Shapley Value (Feature Importance)', fontsize=12)
# 移动顶部的 X 轴，避免与底部 X 轴重叠
ax2.xaxis.set_label_position('top')  # 将标签移动到顶部
ax2.xaxis.tick_top()#将刻度也移动到顶部
# 设置 y 轴标签
ax1.set_ylabel('Feature', fontsize=12)
plt.tight_layout()
plt.savefig("SHAP_combined_with_top_line_corrected.pdf", format='pdf',
bbox_inches='tight')
plt.show()


explainer = shap.TreeExplainer(best_model['rf'])
# 计算训练集和测试集的 SHAP 值
shap_values_train = explainer.shap_values(X_train)
shap_values_X = explainer.shap_values(X)
shap_values_test = explainer.shap_values(X_test)
# 绘制 SHAP 值总结图（Summary Plot）
plt.figure(figsize=(15, 5))
plt.subplot(1, 3, 1)
shap.summary_plot(shap_values_train, X_train,x_columns, plot_type="bar",
show=False)
plt.title("X_train")
plt.xlabel('')  # 移除 x 轴标签避免 x 轴重叠
plt.subplot(1, 3, 2)
shap.summary_plot(shap_values_X,X ,x_columns, plot_type="bar",
show=False)
plt.title("X")
plt.subplot(1, 3, 3)
shap.summary_plot(shap_values_test, X_test,x_columns, plot_type="bar",
show=False)
plt.title("X_test")
plt.xlabel('')
plt.tight_layout()
plt.show()


X_test_=pd.DataFrame(X_test,columns=x_columns)
X_test_.head()

shap.dependence_plot('d-Clavicles', shap_values, X_test_,
interaction_index='∠L-Clavicles')
```

```
# 绘制单个样本的 SHAP 解释（Force Plot）
sample_index = 201   # 选择一个样本索引进行解释
shap.force_plot(explainer.expected_value,   shap_values_test[sample_index],
X_test_.iloc[sample_index], matplotlib=True)


shap_interaction_values = explainer.shap_interaction_values(X_test_)
shap.summary_plot(shap_interaction_values, X_test_)


# 创建 shap.Explanation 对象
shap_explanation = shap.Explanation(values=shap_values[0:500,:],
                                    base_values=explainer.expected_val
ue,
                                    data=X_test_.iloc[0:500,:],
feature_names=X_test_.columns)
# 绘制热图
shap.plots.heatmap(shap_explanation)


# 计算 shap 值为 Explanation 格式
shap_values_Explanation = explainer(X_test_)
shap_values_Explanation
feature_index = shap_values_Explanation.feature_names.index('name')
feature_index
shap_values_Explanation[:, feature_index]


# 指定特征的名称为 ''
feature_name = 'name'
# 找到指定特征的索引
feature_index = shap_values_Explanation.feature_names.index(feature_name)
plt.figure(figsize=(10, 5), dpi=1200)
# 使用 SHAP 的 scatter 方法绘制指定特征的散点图
shap.plots.scatter(shap_values_Explanation[:, feature_index], show=False)
plt.title(f'')
plt.tight_layout()
plt.show()
```
